# Breakers and amplifiers in chromatin circuitry: acetylation and ubiquitination control the heterochromatin machinery

**DOI:** 10.1016/j.sbi.2021.06.012

**Published:** 2021-12

**Authors:** Luke T. Bailey, Sarah J. Northall, Thomas Schalch

**Affiliations:** Leicester Institute for Structural and Chemical Biology, Department of Molecular and Cell Biology, University of Leicester, Leicester, LE1 9HN, UK

## Abstract

Eukaryotic genomes are segregated into active euchromatic and repressed heterochromatic compartments. Gene regulatory networks, chromosomal structures, and genome integrity rely on the timely and locus-specific establishment of active and silent states to protect the genome and provide the basis for cell division and specification of cellular identity. Here, we focus on the mechanisms and molecular machinery that establish heterochromatin in *Schizosaccharomyces pombe* and compare it with *Saccharomyces cerevisiae* and the mammalian polycomb system. We present recent structural and mechanistic evidence, which suggests that histone acetylation protects active transcription by disrupting the positive feedback loops used by the heterochromatin machinery and that H2A and H3 monoubiquitination actively drives heterochromatin, whereas H2B monoubiquitination mobilizes the defenses to quench heterochromatin.

## Highlights

• Heterochromatin-associated complexes are attracted and repelled by histone marks.
• Acetylation of specific lysine residues protects euchromatin from silencing.
• Methylation of histone H3 lysine 9 and 27 amplifies heterochromatin.
• Nucleosome ubiquitination licences and enforces feedback loops.

## Introduction

The accurate and timely organization of the genome into active and inactive genes, domains, and compartments is critical for organism development, for determination of cellular identity, and for genome replication, segregation, and protection [1]. In eukaryotes, chromatin represents both the physiological form of the genome as well as the main signaling platform for coordinating genome function. The histone octamers assemble 146 bp segments of DNA into nucleosomes [2], which cover the genome and occlude DNA from access by the nuclear machinery. To activate gene expression, sequence-specific transcription factors work in concert with co-activators to recruit and provide DNA access to the transcription machinery. In contrast, repressive complexes act to tighten chromatin structure and keep specific genes or entire compartments silent [3]. Chromatin states can be divided into euchromatin, the transcriptionally active regions, and heterochromatin, which replicates late and represses not only transcription but also recombination. Establishing chromatin states in a timely and specific manner is key to maintaining genome stability and transcriptional programs for healthy cell and organism development [4].

Histone post-translational modifications (PTMs) are ‘read, written, and erased’ by chromatin-modifying complexes to establish their foothold on a genomic target and to attract supporting factors [5,6]. In addition to signaling, histone variants like H2A.Z, CENP-A, and PTMs including lysine acetylation and ubiquitination change the biophysical properties of chromatin and contribute directly to genome function [7]. Histone PTMs play an important role in the regulation of the transcriptional gene silencing machinery and in establishing the positive feedback loops that drive the stability of the silent state [8]. On one hand, acetylation is used to ‘open up’ chromatin through loss of the lysine charge, and on the other, acetylation provides signaling via specific histone lysine residues [9]. Here, we will focus on specific acetylation and ubiquitination sites and on the much less appreciated function of histone marks in repelling heterochromatic complexes and breaking of feedback loops.

## Fission yeast heterochromatin relies on a highly regulated web of silencing complexes

**Figure 1:**
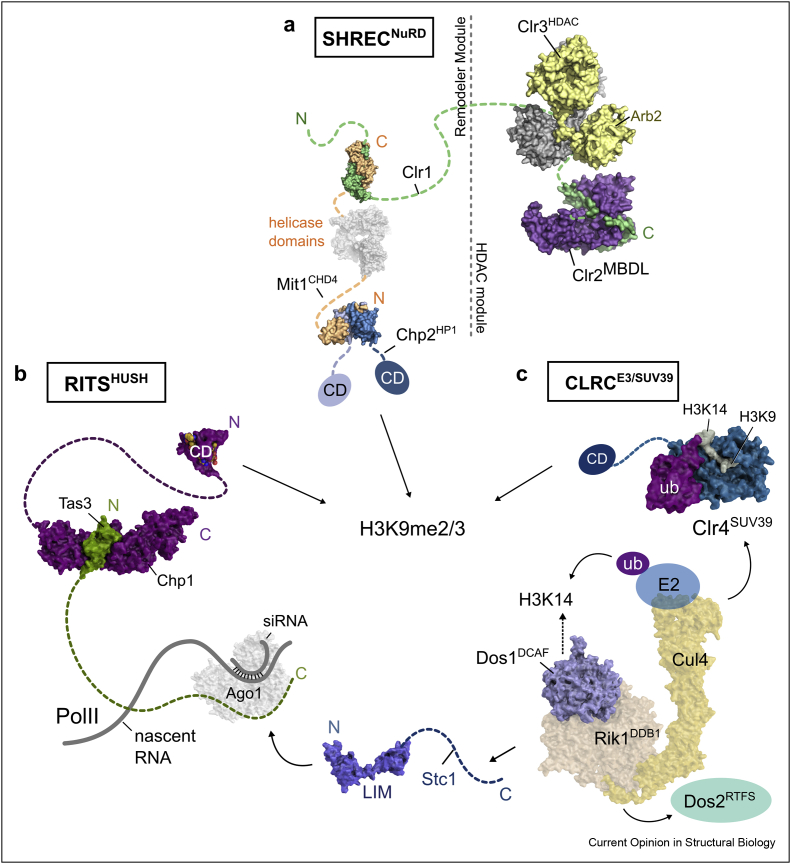
*S. pombe* heterochromatin complexes combine RNAi and chromatin modifiers anchored to the H3K9me2/3 platform. **(**a**)** The model of the RITS complex based on the crystal structures of the Chp1 chromodomain (PDBID:3G7L) and the Chp1-Tas3 subcomplex shows how Ago1 (model) and siRNAs are connected to H3K9me2/3 [17,65]. Although homology with mammalian silencing complexes has long been elusive, recent structural work on the mammalian HUSH silencing complex has revealed a similar architecture to the RITS complex [66]. CD: chromodomain. **(**b**)** The model based on crystal structures of the nucleosome remodeling and deacetylation complex SHREC shows how the building blocks assemble on the Clr1 scaffold into a remodeler module targeted to H3K9me2/3 and an HDAC module guided by the MBD-like Clr2 subunit [26,38]. Arb2: Arb2 domain. **(**c**)** Proposed structure of the CLRC complex, which is closely related to Cul4-ring ligases (CRL4) and targeted by the WD40 protein Dos1 to the ubiquitination target histone H3 lysine 14. Clr4 is a subunit of CLRC and recognizes the ligation product H3K14ub, which strongly stimulates H3K9 methyltransferase activity. However, it remains poorly understood how Clr4 associates with the E3 ligase [23]. LIM: LIM domain.

In the fission yeast *Schizosaccharomyces pombe*, heterochromatin dominates the centromeres, telomeres, and mating-type locus. It depends on the intimate cooperation of highly conserved chromatin modifiers and RNAi complexes, which use small RNAs and histone PTMs to establish silent genomic regions (reviewed in Refs. [10, 11, 12]). Among the most prominent complexes are the RNAi-induced transcriptional gene silencing (RITS) complex, the Snf2/Hdac-containing repressor complex (SHREC), and the Clr4 complex (CLRC) (Fig. 1). They establish a chromatin environment characterized by low histone turnover and hypoacetylation and engage in feedback loops by depositing and reading dimethylated and trimethylated histone H3 lysine 9 (H3K9me2/3) [13, 14, 15]. SHREC belongs to the family of nucleosome remodeling and deacetylation (NuRD) complexes and combines the histone deacetylase (HDAC) Clr3 with the chromatin remodeler Mit1 (Fig. 1a). The RITS complex represents a central node in the *S. pombe* silencing network as it combines small-RNA–guided sequence specificity of Ago1 and the high-affinity sensing of H3K9me2/3 through the chromodomain of Chp1 [16,17] (Fig. 1b). RITS also interacts with CLRC through the Stc1 subunit and thereby tethers CLRC [18,19], which consists of the H3K9 methyltransferase Clr4 and the E3 ubiquitin ligase Rik1 (Fig. 1c). CLRC shows strong similarity to the DDB1 family of Cullin4-RING ligases [20,21] and further contains Dos1 (also known as Raf1), an adapter molecule that guides the E3 ligase to its target, and Dos2 (also known as Raf2), which contains a replication focus targeting sequence. Although the role of Dos1 was shown to be consistent with a DDB1- and CUL4-associated factor [22,23], its target has remained elusive. It is furthermore unclear how Clr4, Dos2, and Stc1 associate with Rik1.

Both the methyltransferase activity of Clr4 and the E3 ligase activity of CLRC are required for the deposition of H3K9me2/3 marks to provide the platform for recruitment of heterochromatin effector proteins [21]. Swi6 is an HP1 homolog and the most abundant chromodomain protein in *S. pombe*. By binding to H3K9m2/3 through its chromodomain and to PxVxL motif-containing proteins through its chromo shadow domain, Swi6 recruits histone deacetylases, cohesin, histone chaperones, and many other proteins critical for establishing a repressive environment in heterochromatin [24]. Chp2, in contrast, is less abundant and tightly and exclusively interacts with SHREC using a motif that diverges significantly from the canonical PxVxL motif [25,26].

## Why does H3K14 have a unique role in heterochromatin formation?

**Figure 2:**
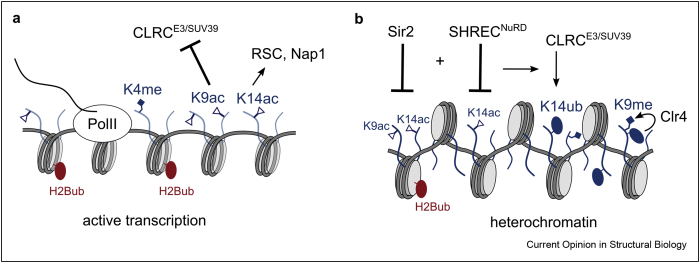
H3K14 is central in the compartmentalization of *S. pombe* chromatin. **(**a**)** The interconnected chromatin marks H2Bub, H3K4me, H3K9ac, and H3K14ac mark loci of active gene transcription due to the full complement of elongation factors that is associated with elongating PolII. H3K9 and H3K14 acetylation directly suppresses heterochromatin formation on actively transcribed genes through inhibition of the CLRC complex. Transparent nucleosomes indicate increased histone turnover by the RSC remodeler and histone chaperones like Nap1, which contributes to maintenance of euchromatin. All tails and modifications in blue refer to H3. **(**b**)** SHREC and Sir2 recruitment is necessary to provide a ‘blank’ template where heterochromatin can form through recruitment of CLRC by RITS and other mechanisms. Licensing of H3K9 methylation by H3K14ub allows for the establishment of heterochromatic feedback loops that rely on the chromodomain proteins of the RITS, SHREC, and CLRC complexes (see Fig. 1).

In *S. pombe*, acetylation of H3K14 (H3K14ac) by the histone acetyltransferases Gcn5 and Mst2 is associated with open reading frames of expressed genes [27,28]. H3K14ac is proposed to play a specific role in recruiting SWI/SNF and RSC chromatin remodeling complexes to provide access to promoter regions [29,30] (Fig. 2a). *In vitro*, H3K14ac is required and sufficient to support nucleosome disassembly by the histone chaperone Nap1 [31]. However, the relevance of these mechanisms for gene expression levels in *S. pombe* is not clear because H3K14ac is enriched on open reading frames and not in promoters. H3K14ac levels are regulated by the zinc-dependent HDAC Clr3, which shows exquisite specificity for H3K14ac, and by the NAD^+^-dependent HDAC Sir2, which deacetylates H3K14ac, H3K9ac, and H4 lysines 5, 12, and 16 [14,32,33]. While deletion of Clr3 or Sir2 alone has a modest effect on gene expression and heterochromatin, simultaneous deletion leads to complete loss of heterochromatin [34,35]. This suggests that the deacetylation of H3K14ac is a key factor in regulating the balance between euchromatin and heterochromatin. This is supported by the H3K14A and H3K14R mutants, which approximately mimic the acetylated and deacetylated states. Intriguingly, both mutations lead to a similar defect in heterochromatin with strong defects in gene silencing and loss of Clr4 and H3K9me2/3 from heterochromatin [34,36,37]. This suggests that the chemical reactivity of the lysine in position 14 is essential for heterochromatin formation, and it suggests that acetylation is an important, but not the only, modification on H3K14 used for signaling in compartmentalizing the genome.

## Clr3 is a highly regulated H3K14-specific HDAC and a subunit of SHREC^NuRD^

How does the Clr3 HDAC achieve this unique specificity for H3K14? The crystal structure of Clr3 revealed a dimeric assembly with both the HDAC domain and the C-terminal Arb2 domain contributing to dimerization and integration into the SHREC^NuRD^ complex [38] (Fig. 1a). The Clr3 HDAC dimer shows very high structural similarity to the tandem HDAC domain structures of mammalian and zebra fish HDAC6, whose tight interaction between the two HDAC domains is critical for full activity [39,40]. Clr3 has a very dynamic active site, which is likely to be formed on binding the acetylated H3 tail. The dimerization and the flexible active site suggest ample opportunities for fine-tuning mechanisms to regulate its activity and specificity.

Immunoprecipitation experiments targeting Clr3 showed that it forms a NuRD-family complex called SHREC together with the remodeler Mit1 [25,41]. SHREC^NuRD^ is recruited for silencing by the HP1 protein Chp2 [24, 25, 26∗] and by the Clr2 subunit, which shows structural similarity to mammalian MBD subunits of NuRD [38] (Fig. 1a). NuRD complexes are recruited through sequence-specific transcription factors in the mammalian system and through the subunits MBD2 and MBD3, which bind methylated CpG with high and low affinity, respectively (reviewed in Ref. [42]). Given that *S. pombe* has no DNA methylation, the mechanism of Clr2-mediated recruitment remains enigmatic. Potential mechanisms might involve Seb1, which recognizes noncoding transcription, and Lem2, a nuclear membrane anchor and interactor of Clr2 [43,44].

In summary, Clr3 is regulated on many levels to specifically deacetylate H3K14ac and is recruited as part of SHREC, but also independently [41,45]. The fact that the double mutant of Sir2 and Clr3 show complete loss of heterochromatin indicates that Clr3 acts upstream of Clr4 and that deacetylation of H3K14ac is critical for heterochromatin formation.

## H3K14 ubiquitination stimulates the H3K9 methyltransferase activity of Clr4

Recently, a new role for H3K14 emerged in heterochromatin silencing. Using the purified CLRC complex, Oya et al. [37] have shown that CLRC's E3 ligase activity preferentially targets histone H3, specifically the H3K14 residue for monoubiquitination, and that compared with the unmodified histone H3 tail, a ubiquitinated H3K14 (H3K14ub) peptide is a much better substrate for H3K9 methyltransferase activity of Clr4. They further demonstrated that H3K14ub can be detected by mass spectrometry on histone tails when enriching for histones carrying the H3K9me2 mark. Our structural investigation of the Clr4 stimulation by H3K14ub has revealed that H3K14ub binds with high affinity to Clr4 and that it furthermore leads to increased enzymatic turnover, suggesting an induced fit mechanism that relays the crosstalk between H3K14ub and H3K9me2/3 (Fig. 2b). Disabling the recognition of H3K14ub by Clr4 through mutagenesis leads to complete loss of heterochromatin [46]. These results suggest a heterochromatic licensing mechanism that controls the deposition of H3K9me2/3 and downstream assembly of functional heterochromatin on a specific region of the genome. Thus, H3K14 becomes a key regulatory switch that controls heterochromatic versus euchromatic states. Acetylation of H3K14 and H3K9 blocks ubiquitination of H3K14 (Fig. 2a) and methylation of H3K9, and therefore, heterochromatin can only form when the combined HAT and HDAC activities result in low acetylation levels and when CLRC is recruited to ubiquitinate H3K14 (Fig. 2b).

While the positive effect of acetylation in gene expression is often explained by its potential to relax chromatin structure due to loss of positive charges on histone tails, the key role of H3K14 in H3K9 methylation shows that specific acetylation sites function to break the feedback loops that drive heterochromatin establishment and maintenance. In the following, we will analyze *Saccharomyces* *cerevisiae* and metazoan polycomb silencing mechanisms to show that the concept of using specific histone lysine residues to protect euchromatin is widely used across highly divergent heterochromatin systems.

## H4K16ac and H3K79me protect from silencing by the Sir complex

**Figure 3:**
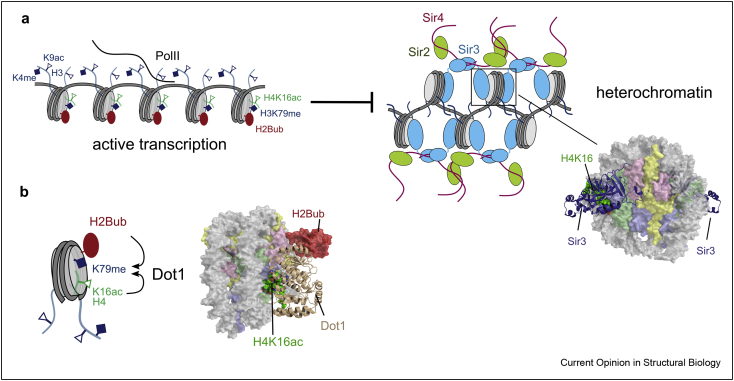
H4K16ac and H3K79me repel Sir3 in *S*. *cerevisiae*: **(**a**)** H4K16ac repels Sir3–nucleosome binding. In heterochromatin, Sir2 deacetylates H4K16ac in an NAD-dependent manner, allowing stable binding of Sir3 to the NCP by the interaction of H4K16 with a negatively charged pocket of Sir3. The Sir3 BAH domain is shown in complex with both sides of the NCP disk surface (PDB: 3TU4) [67]. **(**b**)** Dot1 histone methyltransferase docks onto the nucleosome acidic patch and makes contacts with H2BK123ub and H4K16ac that stimulate SAM-dependent- transfer of a methyl group to generate H3K79me, a marker of euchromatin. Dot1 in complex with the H4K16ac NCP disk surface is shown, (PDB:7K6Q) [50].

H4K16 acetylation (H4K16ac) is a mark tightly associated with active gene transcription and has been shown to reduce the compaction of chromatin fibers [47]. However, H4K16 has also emerged as a critical site for integrating chromatin signaling. *S. cerevisiae* uses the unique set of silent information regulator (SIR) proteins for heterochromatin formation, which form a complex composed of Sir2, Sir3, and Sir4 that binds heterochromatic regions in a finely tuned balance between SIR proteins and the chromatin substrate (reviewed in Ref. [48]). Binding of Sir3 to the nucleosome plays a key role and is negatively affected not only by H4K16ac but also by H3K79 methylation (H3K79me), a PTM on the surface of the folded core of the histone octamer (Fig. 3a). H2B monoubiquitination (H2Bub) controls H3K79me and H3K4me by guiding the methyltransferases Dot1 and Set1 to their substrates [49]. Recent work has revealed in atomic detail how Dot1 integrates both H2Bub and H4K16ac to stimulate H3K79 methylation [50], showing that a methyltransferase like Dot1 is subject to regulation by ubiquitination and acetylation simultaneously (Fig. 3b). H2Bub has also been implicated in heterochromatin protection in *S. pombe*, where acetylation of the H2B ubiquitin ligase subunit Brl1 by Mst2 regulates H2Bub levels and protects euchromatic genes from heterochromatic silencing by small RNAs [51]. H4K16ac in *S. cerevisiae* is a classic example of ‘anti-silencing’, which corrals heterochromatin factors into silent regions of the genome to protect active gene transcription from silencing [52]. Even though *S. cerevisiae* and *S. pombe* use very different heterochromatin machinery, the functional parallels between scH4K16ac and spH3K14ac are becoming apparent and point to a critical role of specific acetyl marks as repellents in the protection of euchromatin. H3K14ac has further been implicated in the protection of euchromatin as it was found to protect H3K4me through inhibition of H3K4 demethylase activity for both human LSD1 and the *S. cerevisiae* JARID1 homolog Jhd2 [53,54].

## Ubiquitination drives the feedback loop in polycomb silencing

**Figure 4:**
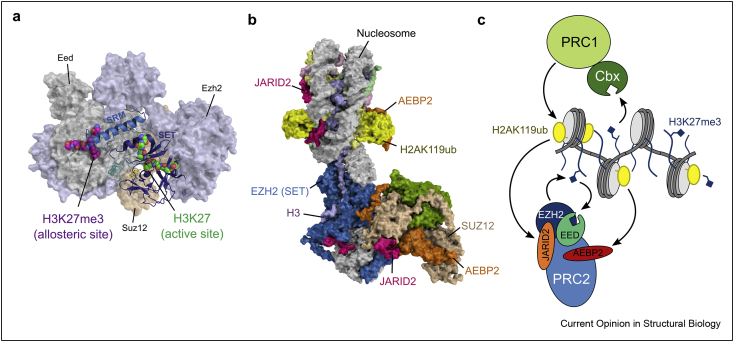
Ubiquitin and H3K27me3 promote polycomb silencing. **(**a**)** The crystal structure of PRC2 (PDBID:5KJH) reveals how the H3K27me3 peptide bound to Eed communicates with the Ezh2 SET domain (blue) through the Suz12 SRM (pink) and SAL (green) domains. This was adapted from Ref. [59]. **(**b**)** H2AK119ub is recognized by the JARID2 and AEBP2 subunits of PRC2 (PDBID:6WKR) [63]. **(**c**)** Feedback between PRC1 and PRC2 drives transcriptional repression. PRC1 deposits H2AK119ub, which, together with H3K27me-mediated stimulation through EED, guides PRC2 H3K27 methyltransferase activity. H3K27 me further recruits PRC1 through the Cbx chromodomain.

Polycomb repressor complexes (PRCs) were discovered as repressors of HOX genes in *Drosophila* development and fall into two distinct subfamilies: the PRC1 complexes are ubiquitin ligases that catalyze the monoubiquitination of H2AK119 (H2AK119ub), whereas PRC2 complexes are H3K27 methyltransferases (reviewed in Ref. [55]). These two families of complexes work together to establish repressed genomic regions during development and are implicated in various cancers [4]. The first identified heterochromatin-enforcing feedback loop between PRC1 and PRC2 complexes was attributed to the chromodomain-containing CBX subunits of PRC1, which bind the H3K27me2/3 marks deposited by PRC2 [56]. It is now clear that PRC2 also binds its own mark using the EED subunit in a positive feedback loop similar to the chromodomain in Clr4 and other SUV39 family H3K9 methyltransferases, thereby facilitating the spreading of facultative heterochromatin in cis [57,58]. Recognition of H3K27 methylation also stimulates the H3K27 methyltransferase activity of the EZH2 SET domain through an allosteric mechanism [59,60] (Fig. 4a).

The mechanism of how histone H2A lysine 119 ubiquitination (H2AK119ub) contributes to PRC silencing has started to emerge recently [61,62], and the cryo-electron microscopy (cryo-EM) structure of PRC2 bound to an H2AK119ub-modified nucleosome has established that the PRC2 subunits JARID2 and AEBP2 bind H2BK119ub and guide EZH2 methylation of H3K27 [63] (Fig. 4b). Thus, depending on subunit composition, PRC complexes can engage in feedback loops that use H2AK119ub and methylated H3K27 (H3K27me) to drive the spreading of PRC complexes, high occupancy on chromatin, and transcriptional repression (Fig. 4c).

In the polycomb system, H3K27 acetylation is in direct competition with H3K27 methylation and protects active transcription from silencing. It has been found that NuRD complexes facilitate the silencing of active genes by deacetylating H3K27ac and thereby preparing the chromatin landscape for polycomb repression [4,64].

## Conclusions

Even though organisms use highly divergent systems to segregate their genomes into heterochromatin and euchromatin, the following fundamental concepts are shared between many systems: (1) Acetylation of specific lysines protects euchromatin from the heterochromatin machinery. (2) HDACs prepare chromatin for the establishment of feedback loops that drive heterochromatin formation. (3) Nucleosome ubiquitination guides and enforces the feedback loops to stabilize heterochromatin and euchromatin. The recently discovered signaling through H3K14ub in fission yeast shows that acetylation not only protects the direct methylation targets of the heterochromatin machinery but that acetylation can also control regulatory crosstalk through ubiquitination. In the mammalian system, these regulatory mechanisms remain much less well understood, and we anticipate that exciting discoveries remain to be made regarding the compartmentalization mechanisms of the genome in cell homeostasis and disease.

## Conflict of interest statement

Nothing declared.
